# Effects of short‐term mild hypercapnia during head‐down tilt on intracranial pressure and ocular structures in healthy human subjects

**DOI:** 10.14814/phy2.13302

**Published:** 2017-06-14

**Authors:** Steven S. Laurie, Gianmarco Vizzeri, Giovanni Taibbi, Connor R. Ferguson, Xiao Hu, Stuart M. C. Lee, Robert Ploutz‐Snyder, Scott M. Smith, Sara R. Zwart, Michael B. Stenger

**Affiliations:** ^1^ KBRwyleHoustonTexas; ^2^ Department of Ophthalmology and Visual SciencesThe University of Texas Medical BranchGalvestonTexas; ^3^ MEI TechnologiesHoustonTexas; ^4^ Department of Physiologic NursingUniversity of California, San FranciscoSan FranciscoCalifornia; ^5^ University of Michigan School of NursingDepartment of Applied Biostatistics LaboratoryAnn ArborMichigan; ^6^ NASA Lyndon B. Johnson Space CenterHoustonTexas; ^7^ Department of Preventive Medicine & Community HealthThe University of Texas Medical BranchGalvestonTexas

**Keywords:** Head‐down tilt, hypercapnia, NASA, one‐carbon metabolism, translaminar pressure difference

## Abstract

Many astronauts experience ocular structural and functional changes during long‐duration spaceflight, including choroidal folds, optic disc edema, globe flattening, optic nerve sheath diameter (ONSD) distension, retinal nerve fiber layer thickening, and decreased visual acuity. The leading hypothesis suggests that weightlessness‐induced cephalad fluid shifts increase intracranial pressure (ICP), which contributes to the ocular structural changes, but elevated ambient CO_2_ levels on the International Space Station may also be a factor. We used the spaceflight analog of 6° head‐down tilt (HDT) to investigate possible mechanisms for ocular changes in eight male subjects during three 1‐h conditions: Seated, HDT, and HDT with 1% inspired CO_2_ (HDT + CO_2_). Noninvasive ICP, intraocular pressure (IOP), translaminar pressure difference (TLPD = IOP‐ICP), cerebral and ocular ultrasound, and optical coherence tomography (OCT) scans of the macula and the optic disc were obtained. Analysis of one‐carbon pathway genetics previously associated with spaceflight‐induced ocular changes was conducted. Relative to Seated, IOP and ICP increased and TLPD decreased during HDT. During HDT + CO_2_ IOP increased relative to HDT, but there was no significant difference in TLPD between the HDT conditions. ONSD and subfoveal choroidal thickness increased during HDT relative to Seated, but there was no difference between HDT and HDT + CO_2_. Visual acuity and ocular structures assessed with OCT imaging did not change across conditions. Genetic polymorphisms were associated with differences in IOP, ICP, and end‐tidal PCO_2_. In conclusion, acute exposure to mild hypercapnia during HDT did not augment cardiovascular outcomes, ICP, or TLPD relative to the HDT condition.

## Introduction

Astronauts completing long‐duration spaceflight of up to ~6 months have developed ocular structural and functional changes including choroidal folds, optic disc edema, globe flattening, optic nerve sheath distension, retinal nerve fiber layer (RNFL) thickening, and visual acuity decrements (Mader et al. 2011; Alexander et al. 2012), although not all symptoms have developed in all crewmembers. The initial report of these findings in 2011 suggested that postflight lumbar puncture opening pressures were slightly elevated based on measurements in five of six astronauts who developed optic disc edema, although no preflight measurements were available for comparison (Mader et al. 2011). Thus, it was hypothesized that increased intracranial pressure (ICP) may be responsible for the above ocular findings, perhaps secondary to the weightlessness‐induced headward fluid shift, although ICP has never been measured in human subjects during spaceflight.

The spaceflight analog of 6° head‐down tilt (HDT) causes a headward fluid shift similar to that which occurs during spaceflight (Blomqvist et al. 1980; Lathers et al. 1990; Pavy‐Le Traon et al. 2007), but frank optic disc edema, choroidal folds, or decreased near visual acuity have not developed during prolonged bed rest (Taibbi et al. 2013, 2014, 2016), suggesting additional factors from the spaceflight environment may be necessary for ocular changes to develop. Another factor hypothesized to contribute to the development of ocular changes is exposure to the mildly elevated ambient partial pressure of carbon dioxide (PCO_2_) that occurs on the International Space Station (ISS) (Law et al. 2010, 2014). Elevated arterial CO_2_ is a strong vasodilator, and from 2001 to 2012, the range of mean 24‐h ambient PCO_2_ on the ISS was 1.0–5.8 mmHg, with a range in peak 24‐h levels of 1.2–8.3 mmHg. The astronauts described in the first case report of ocular changes flew long‐duration missions to the ISS during this period (Mader et al. 2011). Since 2010, efforts have been made to keep the average 24‐h PCO_2_ below 4 mmHg (Law et al. 2014), yet ocular structural and functional findings continue to develop. While these low levels are unlikely to cause symptoms of CO_2_ toxicity in normal ambulatory subjects on the ground, correlations between 7‐day average CO_2_ levels on the ISS and the predicted probability of headaches suggest that weightlessness may increase the sensitivity to mild elevations in CO_2_ (Law et al. 2014). Thus, the combination of a headward fluid shift with mild elevations in CO_2_ may provide a synergistic stimulus that increases cerebrovascular blood flow and elevates ICP (Sofronova et al. 2015), resulting in an imbalance of fluid pressures at the optic disc and decreases the translaminar pressure difference (TLPD, intraocular pressure [IOP] ‐ ICP). Prolonged exposure to a lowered TLPD may be a factor in developing the ocular structural changes observed during spaceflight. While the etiology of spaceflight‐induced ocular changes remains unknown, no studies have linked mild hypercapnia in combination with a headward fluid shift with physiological responses that, if maintained for three to six months, could produce ocular structural and functional changes.

Given that not all astronauts develop ocular changes and that individual variability exists in symptom severity, it is likely that some predisposing factors confer greater susceptibility to developing ocular changes in certain astronauts. Genetic differences in the one‐carbon metabolic pathway had been hypothesized to be associated with the development of ophthalmologic changes during spaceflight (Zwart et al. 2012), and recent evidence documents an association between single‐nucleotide polymorphisms (SNPs) involved in one‐carbon metabolism and choroidal folds, optic disc edema, and cotton‐wool spots (Zwart et al. 2016). Specifically, of the 49 astronauts studied, the minor G allele on the MTRR 66 gene was more prevalent among those that developed choroidal folds or cotton wool spots, whereas the major C allele for gene SHMT1 1420 was associated with optic disc edema. Furthermore, block regression modeling showed that genetics (the number of risk alleles for each gene) and B‐vitamin status were significant predictors of choroidal folds and the degree of visual acuity changes after flight (Zwart et al. 2016).

The purpose of this study was to determine whether acute exposure to mild hypercapnia combined with a cephalad fluid shift induced by HDT would increase cerebral or ocular blood flow, result in an increase in ICP, a reduction in TLPD, and a mild accumulation of fluid at the optic nerve. Given that evidence suggests certain individuals may be more susceptible to ocular changes during spaceflight, we also investigated genetic and biochemical factors associated with one‐carbon metabolism to determine if individuals with similar risk alleles demonstrated greater responses to mild hypercapnia than those with fewer alleles.

## Materials and Methods

### Subjects

The NASA Johnson Space Center Institutional Review Board reviewed and approved this human subject protocol and all methods adhered to the principles outlined in the Declaration of Helsinki. After completing a modified Air Force Class III physical exam and being cleared by the NASA Test Subject Screening facility, eight male subjects provided their written informed consent to participate. Subjects were 35 year old (range: 25–49 year), 181 cm tall (range: 170–193 cm), and weighed 86 kg (range: 73–95 kg).

### Study protocol

All subjects reported to the NASA Johnson Space Center Cardiovascular & Vision Laboratory for a single visit between 0700 and 0800 for a fasted blood draw. Subjects were fitted with a facemask to control inspired gases and for the sampling of expired CO_2_, a continuous finger blood pressure monitor, 3‐lead ECG, and with a headband holding the transcranial Doppler probe. These data were recorded continuously using a data acquisition system (Notochord) for later offline analysis.

**Figure 1:**
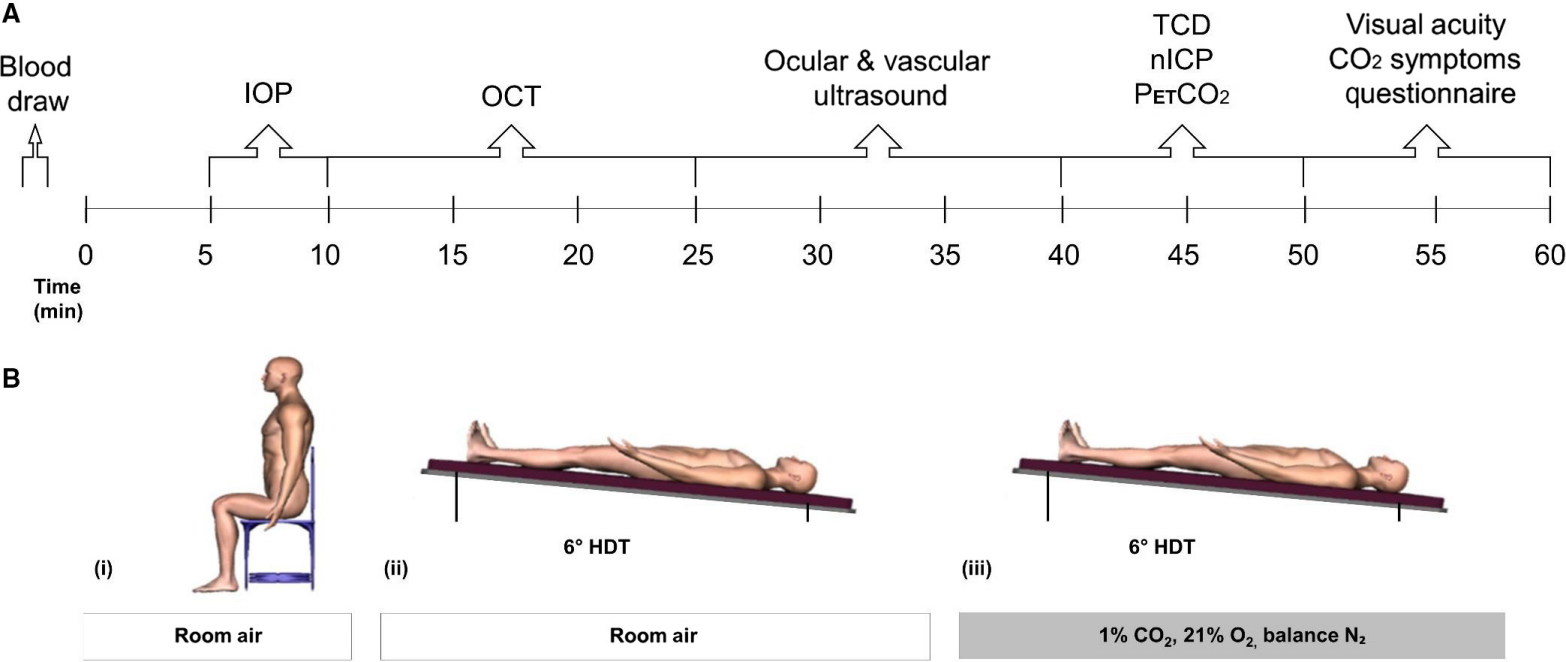
Schematic of study protocol depicting (A) the timeline and order in which measurements were obtained during each condition and (B) the (i) Seated, (ii) HDT, and (iii) HDT + CO_2_ conditions. IOP, intraocular pressure; OCT, optical coherence tomography; TCD, transcranial Doppler ultrasound; nICP, noninvasive measure of intracranial pressure; P_ET_CO_2_, end‐tidal partial pressure of carbon dioxide.

Thereafter, subjects were studied for 60 min each in the following conditions: (A) seated upright breathing room air (Seated); (B) 6° HDT breathing room air (HDT); and (C) 6° HDT breathing 1% CO_2_, 21% O_2_, balance N_2_ (HDT + CO_2_). All subjects completed the Seated condition first, but the order of the two HDT conditions, HDT and HDT + CO_2_, was randomized and balanced. Each condition began with a 5 min stabilization period before data were collected in the following order: ocular tonometry, optical coherence tomography (OCT), ocular and cardiovascular ultrasound, partial pressure of end‐tidal CO_2_ (P_ET_CO_2_), near visual acuity, and completion of a CO_2_ symptom questionnaire (Fig. 1).

### Ocular tonometry

An Icare Pro (Icare, Espoo, Finland) rebound tonometer was used to measure intraocular pressure (IOP). Data collected using this instrument has shown good agreement with data obtained with the Goldmann applanation tonometer, the clinical gold standard for IOP measurement (Moreno‐Montañés et al. 2015). A trained technician obtained three successive IOP measurements per eye starting with the right eye. Each of the three measurements was the result of six successive trials, of which the highest and lowest values were dropped and the remaining four were averaged by the Icare device.

### Optical coherence tomography

Spectralis HRA + OCT (Heidelberg Engineering, Heidelberg, Germany) was used to obtain bilateral OCT scans in High‐Resolution mode. The scanning head was mounted on a surgical arm to obtain OCT scans in the seated and head‐down tilt conditions. The left eye of each subject was scanned first, followed by the right eye. In each eye, the AutoRescan feature was used to anatomically place follow‐up scans in the same location as the baseline (Seated) condition. The built‐in Anatomic Positioning System (APS) was used to obtain optic disc scans aligned to the fovea‐to‐Bruch's membrane opening (BMO) center axis to adjust for head‐tilting and ocular cyclotorsion across consecutive scans from the same eye and ensure consistent sector analysis over time. Specifically, the radial 24‐line scanning pattern aligned to the BMO center was used to obtain the average BMO‐minimum rim width (BMO‐MRW), defined as the shortest linear distance between the BMO and the internal limiting membrane (Povazay et al. 2007; Chen 2009; Chauhan and Burgoyne 2013). The circular scanning pattern, placed evenly around the BMO center, was used to obtain the average RNFL thickness. The volume scanning pattern, centered to the fovea (25 B‐scans over a 20°×20° area), was used to obtain macular thickness.

The Enhanced Depth Imaging line scanning pattern, aligned to the fovea‐to‐BMO center axis, was used to measure choroidal thickness. Choroidal thickness was calculated as the distance between Bruch's membrane, as automatically delineated by Spectralis OCT + HRA software (Heidelberg Eye Explorer, v1.9.10), and the choroidal‐scleral border, as manually delineated by two independent observers. Custom MATLAB scripts determined the mean choroidal thickness over a 3‐mm subfoveal distance. We failed to acquire the line scan in one eye of one subject. Each scan was read by two observers and both measurements of choroid thickness were considered for statistical analysis from the remaining 45 images.

### Ultrasound

Ocular, cardiac, and vascular ultrasound imaging were performed using a GE Vivid Q (Milwaukee, WI) during a 10–15 min period before the MCA velocity data were recorded. Optic nerve sheath diameter (ONSD), ocular axial length, and central retinal artery blood flow velocity (CRA_vel_) were obtained using a 12‐MHz probe with a mechanical index set not to exceed 0.24, in accordance with FDA guidelines. To account for the different hydrostatic fluid column pressure in the central retinal artery between the Seated and HDT conditions, central retinal artery blood flow conductance index (CRAC_i_) was calculated as

$${\text{CRAC}}_{i}=\frac{{\text{CRA}}_{\mathrm{vel}}}{\text{MAP}+\left(\rho gh*0.0075\right)}$$

where *MAP* is the mean brachial arterial pressure, *ρ* is the density of blood (1060 kg·m^−3^), *g* is the acceleration due to gravity (9.8 m·sec^−2^), *h* is the height difference between the brachial artery and the central retinal artery, and 0.0075 converts Pascals to mmHg. Thus, differences in CRAC_i_ between test conditions reflect changes in vascular reactivity rather than changes in arterial pressure resulting from a different hydrostatic column, which influences CRA_vel_.

The same 12‐MHz probe was used to measure the common carotid artery diastolic diameter and flow velocity in order to calculate carotid artery blood flow. A 4‐MHz phased array probe was used to obtain images of left ventricular outflow waveforms (apical window) and aortic root diameter (parasternal long axis). Stroke volume was calculated as the product of the aortic root area and the velocity time integral. All images were stored for offline analysis using Echopac software (Milwaukee, WI). Three representative ultrasound images were analyzed by at least two sonographers blinded to each other's results to verify <10% difference between observers.

### Transcranial doppler

A pulsed Doppler ultrasound probe (Multigon Industries, Elmsford, NY, software version 1.3.1) was fixed in place over the right MCA to measure blood flow velocity with a 1.6‐MHz probe. The probe was held in place over the right temporal window by a headset for the duration of the test, and the MCA was imaged at a depth of about 5 cm. The automated tracing of the peak MCA velocity was recorded by a data acquisition system (Notocord‐hem, Notocord Inc., Newark, NJ) at 250 Hz, which simultaneously recorded the ECG and Finapres signals. Heart rate was monitored with a 3‐lead ECG (Spacelabs 90621A ECG, Spacelabs Inc., Redmond, WA), continuous arterial pressure was monitored using finger photoplethysmography (Finapres, Ohmeda Medical, Amsterdam, Netherlands), and brachial blood pressure (Dinamap XL) was used to estimate mean arterial pressure (MAP). The MCA blood flow velocity was evaluated as a stand‐alone variable and also used in the nICP estimation.

### Noninvasive intracranial pressure

The estimate of noninvasive intracranial pressure (nICP) was modeled with data collected during a 10‐min period of quiet rest starting ~40 min into each condition, using cerebral blood flow velocity (CBFV), finger photoplethysmography (Finapres), and the ECG. The nICP estimation framework (Hu et al. 2010) aims to select an optimal model from a prebuilt database of linear dynamic models of arterial blood pressure (ABP), CBFV, and ICP to simulate ICP using ABP and CBFV from a de novo subject. This approach has been enhanced by a novel machine‐learning algorithm to improve the accuracy of finding the optimal model from the database. In this work, the database contained 169 models that were identified from continuous ABP, CBFV, and invasive ICP signals of 69 brain injury and hydrocephalus patients, as used in our previous work (Kim et al. 2013). The optimal model was chosen for the HDT condition because the subject positioning was most similar to the supine position of patients during data collection used to develop the model database; the same model was applied to the Seated and HDT + CO_2_ conditions, which were masked during analysis. The 10‐min recording was first sectioned into consecutive overlapping segments of 360 heartbeats with 80% overlap, which is equal to the length of the data used in identifying ICP simulation models. Then a continuous ICP signal was estimated for each segment and its mean value was calculated to represent the nICP measurement. Translaminar pressure difference was calculated for each eye as the difference between IOP and nICP (Jonas et al. 2015b). Data from both eyes were used in our statistical model, parameterized as a random nested data within subjects.

### End‐tidal CO_2_

A two‐way non‐rebreathing valve was attached to a facemask (V2 Vacumed, CA) sealed over the nose and mouth and a gas sampling line was attached just beyond the mouth and directed to a rapid‐response CO_2_ gas analyzer (Vacumed, Silver Series). The peaks and troughs recorded from the 30–40 min time point during each condition were analyzed to estimate P_ET_CO_2_ and inspired PCO_2_ (P_I_CO_2_), respectively.

### Near visual acuity

Two near‐vision Early Treatment Diabetic Retinopathy Study Charts were used to assess uncorrected visual acuity of the right and left eyes separately at the end of each of the three conditions.

### CO_2_ symptoms

Subjects were provided a list of 24 symptoms associated with high CO_2_ exposure. This list is used by NASA flight surgeons during crew training after a CO_2_ rebreathing protocol for identification of symptoms related to high CO_2_ exposure. At the end of each condition, subjects rated the intensity of each symptom using a 0–10 scale, with 0 representing no symptoms and 10 representing severe symptoms.

### One‐carbon genetics and biochemistry

Fasted blood samples were collected from an antecubital vein before all other testing. Within 30 min of collection, serum was separated by centrifugation and aliquots were stored at −80°C until batch analysis was done at the end of the study. One EDTA tube was frozen intact and transferred to the University of Florida Clinical and Translational Science Institute's Genotyping Core (Gainesville, FL). Five SNPs were analyzed using TaqMan allelic discrimination (System 7900; Life Technologies Applied Biosystems, Foster City, CA), and were the same as previously reported (Zwart et al. 2016).

Serum vitamin B12, folate, and red blood cell folate concentrations were analyzed using a commercially available radioimmunoassay (DiaSorin, Stillwater, MN). Methylmalonic acid, 2‐methylcitric acid, homocysteine, and cystathionine were measured by gas chromatography‐mass spectrometry (Metabolite Laboratories, Denver, CO). Pyridoxal 5′‐phosphate and 4‐pyridoxic acid were measured by high‐performance liquid chromatography as previously described (Smith et al. 2005).

### Statistics

All statistical analyses were performed using Stata/IC software (v 14.1, StataCorp LP, College Station, TX). All statistical assumptions were tested before results were interpreted. The data from all of our outcomes met the distributional requirements for the techniques employed without requiring data transformation or nonlinear modeling. Our experimental design exposed one group of *n* = 8 subjects to each of three different conditions (Seated, HDT, and HDT + CO_2_) with ~10‐min washout periods between experimental conditions. Outcomes were collected under all three conditions as described above, providing a completely within‐subject experimental design. Ocular outcomes were collected for right and left eyes and were nested within the statistical model to derive a single value for each variable. P_ET_CO_2_ from each of the ten 1‐min averages were entered into the statistical model as repeated measures, with appropriate parameterization to incorporate within‐subject variability. We submitted the repeated‐measures data to separate mixed‐effects linear regression models (one per outcome) that included fixed parameters enabling estimation of means and confidence intervals, and statistical comparison between conditions. We included random intercept terms to accommodate the nesting of observations within subjects; for ocular measures that were measured in the two eyes, we included an additional random intercept to accommodate random variability between left and right eyes. Means and 95% confidence intervals for each variable are reported in figures and tables, and hypothesis testing was set to a two‐tailed probability to reject the null hypothesis at alpha = 0.05.

In light of the statistically significant associations reported previously between alleles of the SHMT 1420 and MTRR 66 genes and spaceflight‐induced ophthalmic changes (Zwart et al. 2016), we placed our subjects in two groups: subjects in whom both genes expressed alleles previously associated with ophthalmic signs and/or symptoms (MTRR 66 AG or GG and SHMT 1420 CC) were classified as SNP+ (*n* = 4), whereas subjects in whom only 1 gene or no genes had alleles previously associated with ocular changes were classified as SNP− (*n* = 4). Mixed‐effects linear regression models were conducted on select variables in which to compare the two‐way interaction between group and condition and to determine whether SNP+ and SNP− groups responded differently to our experimental conditions.

Acting on the basis of block regression findings from our previous study that indicated genetics (MTRR and SHMT SNPs) and B‐vitamin status predicted choroidal folds and visual acuity changes after flight (Zwart et al. 2016), we conducted a multiple regression analysis to determine whether the number of risk alleles for MTRR and vitamin B12 status predicted P_ET_CO_2_ during the HDT + CO_2_ condition. Other factors used in the previously reported model (e.g., age, additional B‐vitamins, other SNPs) could not be included because of limitations in the sample size and data set. Student's *t*‐tests were used to compare the B‐vitamin status of the SNP+ and SNP− groups.

## Results

**Figure 2:**
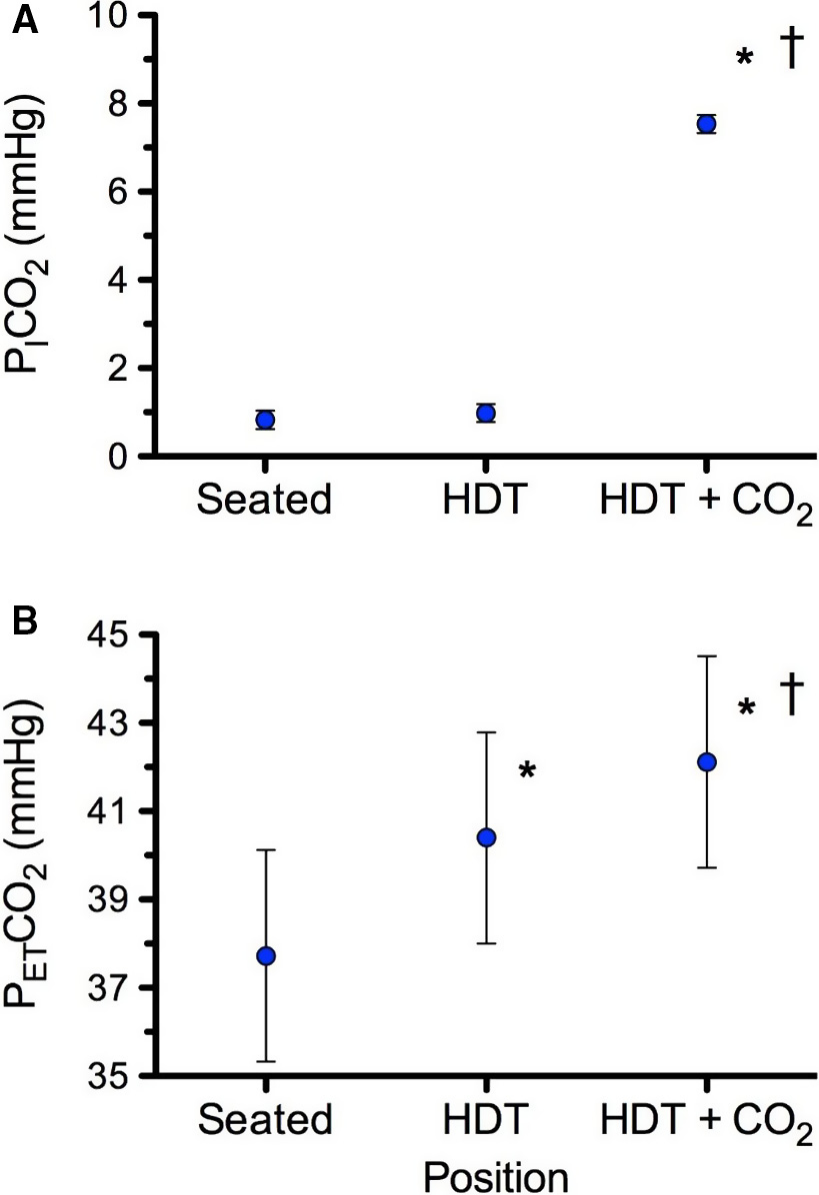
(A) Inspired PCO_2_ (P_I_CO_2_) and (B) end‐tidal PCO_2_ (P_ET_CO_2_) for all subjects in each of the three conditions. Values are means ± 95% confidence intervals. *P < 0.05 versus Seated, ^†^P < 0.05 versus HDT. n = 8 subjects for all variables.

All subjects tolerated the three conditions well and reported no adverse effects. Room air and the premixed gas mixture were successfully delivered via a facemask as reflected in the P_I_CO_2_ and the resulting P_ET_CO_2_ for all subjects (Fig 2). Seated P_ET_CO_2_ was 37.7 mmHg, and HDT caused an increase of 2.7 mmHg. The addition of 1% CO_2_ during HDT caused a further increase of 1.7 mmHg to reach a mean P_ET_CO_2_ of 42.1 mmHg.

### Cardiovascular effects

**Figure 3:**
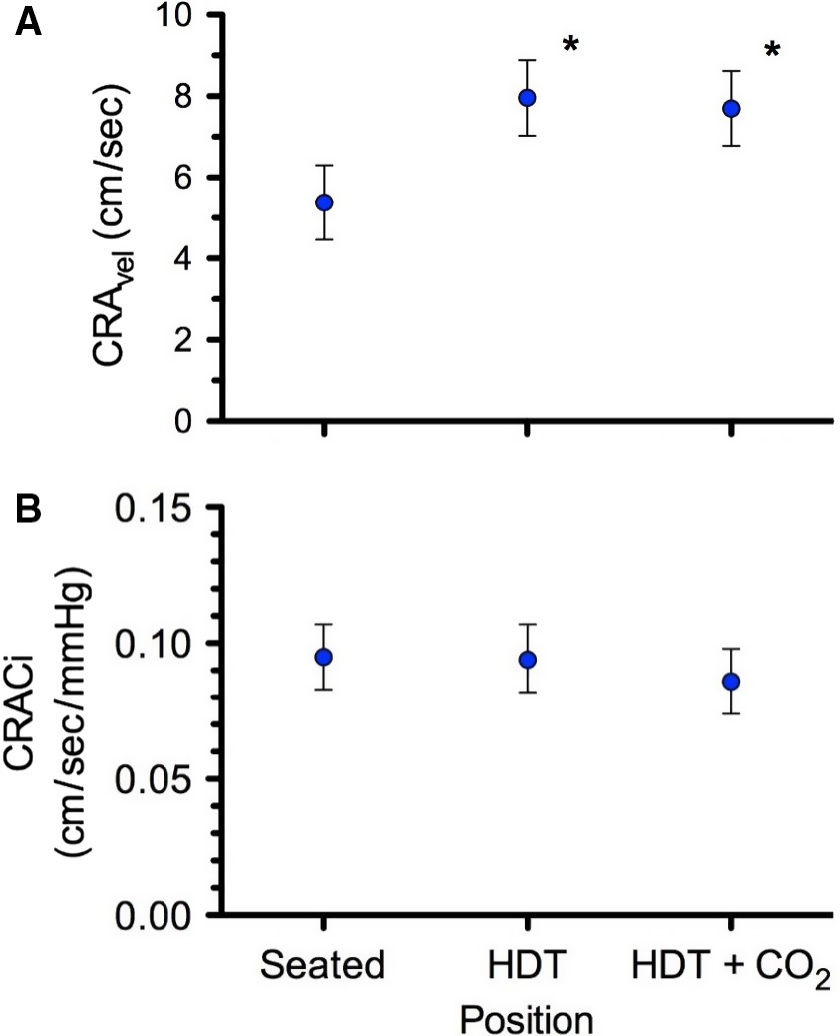
(A) Central retinal artery blood flow velocity (CRA_vel_) and (B) central retinal artery blood flow conductance index (CRAC_i_) for all subjects in each of the three conditions. Values are means ± 95% confidence intervals. *P < 0.05 versus Seated. n = 8 subjects for all variables.

Hemodynamic variables for the three conditions are presented in Table 1. Stroke volume increased and heart rate (HR) decreased from Seated to HDT, but there was no significant difference between the two HDT conditions. Together, these responses resulted in no change in cardiac output or mean arterial pressure (MAP) across the three conditions. Pulse pressure increased during both HDT conditions, which were not different from each other. Common carotid artery blood flow decreased during HDT, but this was driven by the decreased HR. MCA_v_ and CRA_v_ increased during HDT, but did not increase further during HDT + CO_2_. When the change in central retinal arterial pressure during HDT due to the change in fluid column pressure was accounted for using CRAC_i_, there was no difference across the three conditions (Fig. 3). Choroid thickness increased significantly with HDT and HDT + CO_2_, but there were no significant differences between the two HDT conditions (Table 2).

**Table 1:** Hemodynamic variables

Variable	Seated	HDT	HDT+CO_2_
Stroke volume, mL	81.9 (71.3–92.6)	101.2 (90.5–111.9)^a^	102.5 (91.8–113.1)^a^
Heart rate, bpm	58 (52–65)	48.3 (42–55)^a^	48 (41–55)^a^
Cardiac output, mL·min^−1^	4631 (4354–4910)	4816 (4539–5095)	4809 (4532–5088)
Mean arterial pressure, mmHg	89 (82–96)	87 (80–94)	88 (80–95)
Systolic blood pressure, mmHg	116 (108–125)	124 (115–133)	123 (115–132)
Diastolic blood pressure, mmHg	73 (66–81)	69 (61–76)	72 (64–79)
Pulse pressure, mmHg	43 (36–51)	55.5 (48–63)^a^	51.4 (44–59)^a^
Common carotid artery flow, mL·min^−1^	680.8 (605.5–756.0)	630.53 (555.3–705.7)^a^	669.3 (594.1–744.5)
Mean MCA_v_, cm·sec^−1^	52.2 (45.63–58.70)	62 (55.51–68.58)^a^	62 (55.55–68.62)^a^
Mean MCA_v_, % change from seated	–	20.1 (12.1–28.2)	19.7 (11.6–27.7)

HDT, head‐down tilt; MCA, middle cerebral artery. Values are mean (95% CI).

a *P *<* *0.05 versus Seated. There were no significant differences between HDT and HDT + CO_2_.

**Table 2:** Ocular variables

Variable	Seated	HDT	HDT+CO_2_
Central Macular Thickness, *μ*m	274.2 (265.6–282.8)	273.4 (264.7–282.0)	274.1 (265.5–282.8)
Average RNFL Thickness, *μ*m	104.4 (97.1–111.7)	103.9 (96.6–111.2)	103.5 (96.2–110.8)
BMO area, mm^2^	2.09 (1.93–2.25)	2.11 (1.95–2.27)^a^	2.09 (1.93–2.26)
BMO‐MRW, *μ*m	365.1 (337.3–392.9)	363.8 (336.0–391.6)	362.7 (334.9–390.5)
Axial Length, mm	24.44 (23.42–25.46)	24.52 (23.50–25.54)	24.54 (23.52–25.57)
ONSD, mm	6.23 (5.71–6.75)	6.58 (6.06–7.10)^a^	6.66 (6.14–7.18)^a^
Choroid Thickness, *μ*m	348.1 (291.1–405.2)	361 (304.0–418.0)^a^	356.7 (299.6–413.7)^a^
Visual Acuity, logMAR	0.10 (−0.11−0.31)	0.14 (−0.06−0.35)	0.09 (−0.12−0.30)

BMO, Bruch's membrane opening; MRW, minimum rim width; ONSD, optic nerve sheath diameter; RNFL, retinal nerve fiber layer. Values are mean (95% CI).

a *P* < 0.05 versus Seated. There were no significant differences between HDT and HDT + CO_2_.

### IOP, nICP, TLPD

**Figure 4:**
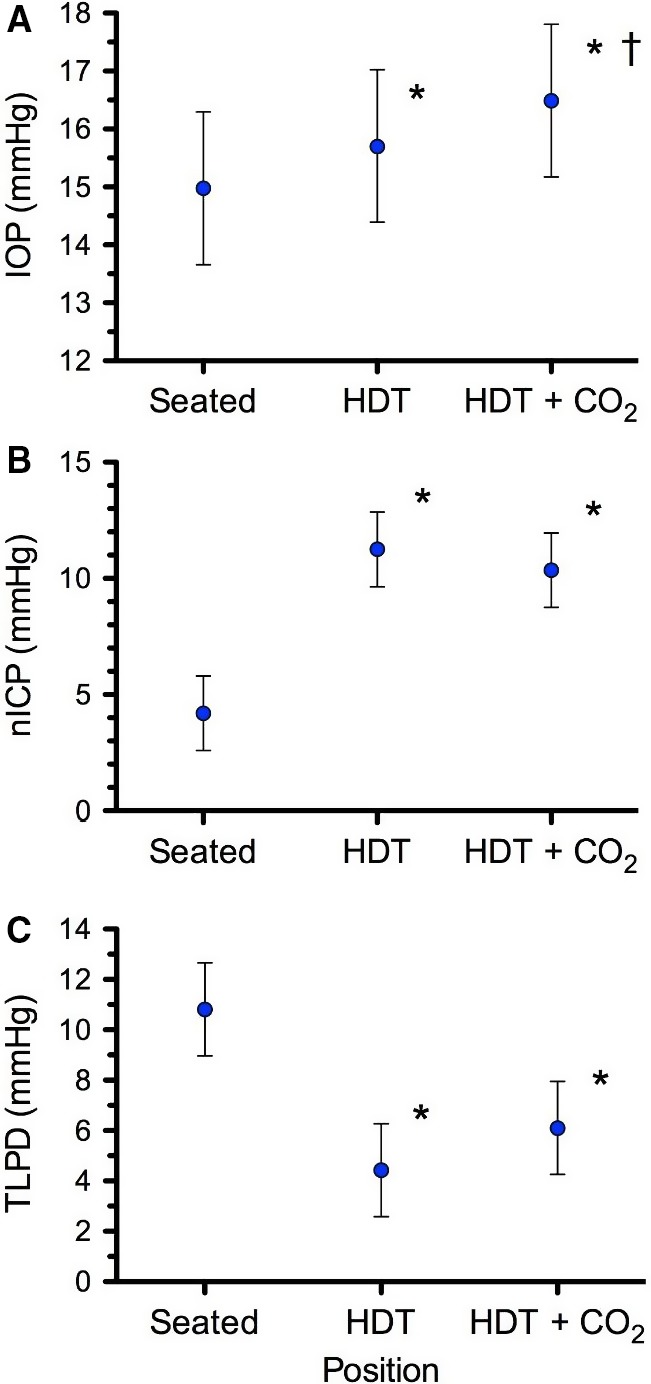
(A) Intraocular pressure (IOP), (B) noninvasive estimate of intracranial pressure (nICP), and (C) the resulting translaminar pressure difference (TLPD = IOP–nICP) for all subjects in each of the three conditions. Values are means ± 95% confidence intervals. *P < 0.05 versus Seated, ^†^P < 0.05 versus HDT. n = 8 subjects for all variables.

HDT caused a small, but significant increase in IOP from 15.0 to 15.7 mmHg, and the addition of 1% CO_2_ significantly increased IOP further to 16.5 mmHg, although all these values were within normal limits (Fig. 4A). Compared to Seated, HDT increased nICP from 4.2 to 11.3 mmHg, but the addition of CO_2_ did not increase it further (Fig. 4B). TLPD was 10.8 mmHg while Seated and decreased to 4.4 mmHg and 6.1 mmHg during HDT and HDT + CO_2_, respectively, which were not different from each other (Fig 4C).

### Ocular structure

Neither HDT, nor HDT+CO_2_ caused any significant changes from baseline in macular thickness, average RNFL thickness, average BMO‐MRW, or axial length (Table 2). The statistically significant increase in BMO area during HDT was likely not physiologically significant. ONSD increased during HDT, but did not increase further during HDT + CO_2_.

### Visual acuity and CO_2_ symptoms

Relative to Seated, there were no changes in visual acuity (Table 2) and no differences in CO_2_ symptoms were observed between HDT and HDT + CO_2_. The highest score for any CO_2_ symptom during HDT + CO_2_ was 1, corresponding to the low end of the mild symptom range (mild = 1–3).

### Genetics and one‐carbon biochemistry

The proportion of subjects bearing the GG allele of the MTRR 66 gene was 25% in this study and 20% among 41 male astronauts (Table 3). Similar proportions were observed between the current population and the astronaut population for the AG allele on the MTRR 66 gene (50% for both) and CC allele for SHMT 1420 gene (63% for this study and 61% for the astronaut male population). The C allele for SHMT and the G allele for MTRR 66 were categorized as the risk alleles (Zwart et al. 2016).

**Table 3:** SNP allele incidence

	HDT + CO_2_ study (*n* = 8 males)			Astronaut data (*n* = 49 total, 41 males)		
	Homozygous (minor allele)	Heterozygous	Homozygous (major allele)	Homozygous (minor allele)	Heterozygous	Homozygous (major allele)
MTRR A66G	2	4	2	10	25	13
MTHFR A1298C	0	3	5	6	16	27
MTHFR C677T	1	5	2	6	19	24
SHMT C1420T	1	2	5	2	16	30
CBS 844ins68	0	0	8	0	8	41

Allele incidence of the eight subjects in this study, along with astronaut data adapted from (Zwart et al. 2016).

There were no statistically significant differences in B‐vitamin status between SNP+ and SNP– (Table 4). In general, B‐vitamin status of our subjects was similar to those of the astronauts on the day of their SNP analysis (Table 4 and (Zwart et al. 2016)).

**Table 4:** Vitamin‐B status

	SNP+ (*n* = 4)	SNP− (*n* = 4)	Astronaut data (*n* = 49)	Normal range
Vitamin B12, pmol/L	416 (165–666)	300 (131–468)	557 (497–617)	150–700
Serum Folate, nmol/L	30 (6–54)	36 (24–48)	33 (27–39)	21–64
Red Blood Cell Folate, ng/mL	485 (195–776)	720 (6–1433)	447 (415–479)	365–1043
Methylmalonic Acid, nmol/L	199 (77–321)	137 (107–167)	201 (177–225)	73–271
2‐Methylcitric Acid, nmol/L	141 (64–218)	142 (90–194)	157 (146–168)	60–228
Total Homocysteine, *μ*mol/L	9 (7.8–10.4)	8 (5.7–9.6)	8 (7.6–8.4)	5.1–13.9
Cystathionine, nmol/L	159 (19–298)	140 (78–201)	175 (153–197)	44–342
Pyridoxal 5′‐phosphate, nmol/L	85 (49–121)	63 (27–98)	108 (83–133)	11–302
4‐pyridoxic acid, nmol/L	16 (4–28)	22 (0–50)	45 (28–62)	9–385

B‐vitamin status by one‐carbon genetics (SNP+, SNP^−^), in test subjects and astronauts. Astronaut data represent status at the time of blood collection for the 1C Study (Zwart et al. 2016) genetic analysis, and were not collected with any specific reference to flight.

**Figure 5:**
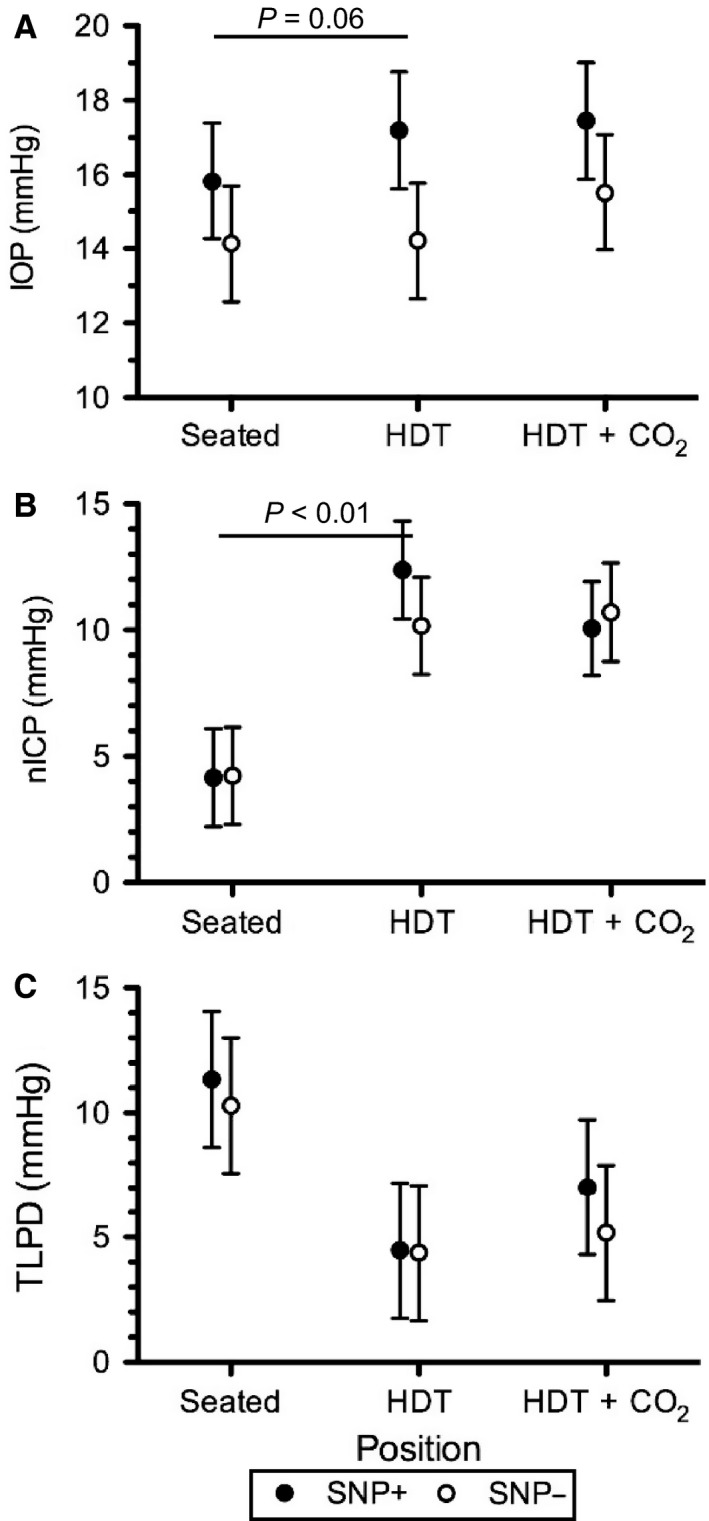
(A) IOP, (B) nICP, and (C) TLPD for SNP+ (filled) and SNP− (open) groups during each condition. Values are means ± 95% confidence intervals. P‐value indicates group by condition interaction. n = 4 subjects in each SNP+ and SNP‐ groups.

When subjects were grouped according to their genetic polymorphisms, interaction effects between the change in position (i.e., Seated to HDT and HDT + CO_2_) and genetic group (i.e., SNP+ vs. SNP–) showed a trend (*P *=* *0.06) toward significance for the IOP response to be larger in SNP+ subjects (Fig. 5A) and a significantly greater increase for nICP in SNP+ subjects (Fig. 5B), but no significant interaction in the TLPD response to either HDT condition (Fig. 5C). The interactive effects for genetic group and the change in position from Seated to HDT + CO_2_ were not statistically significant.

**Figure 6:**
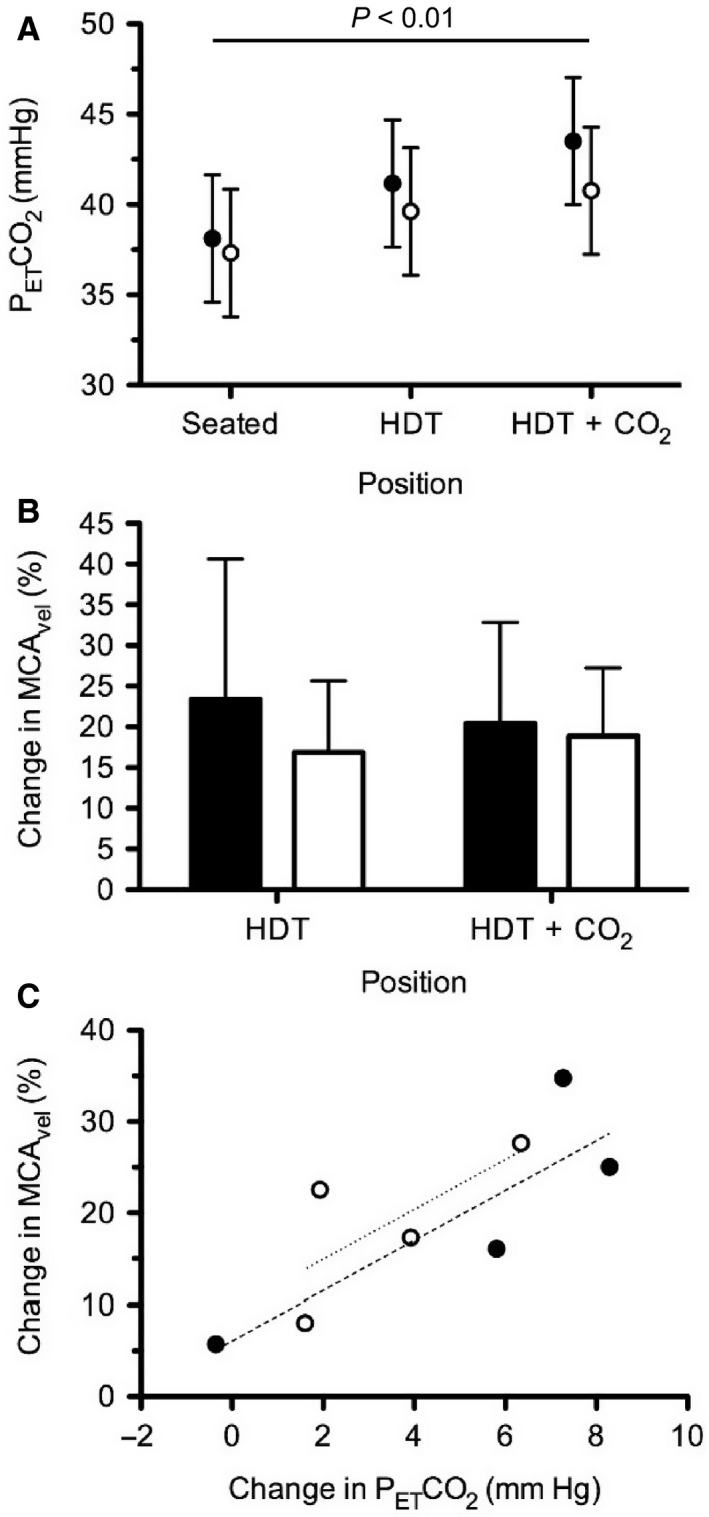
(A) P_ET_CO_2_ for SNP+ (filled) and SNP− (open) groups during each condition. (B) Change in MCA_vel_ compared to Seated for SNP+ (filled, n = 4) and SNP− (open, n = 4) groups during head‐down tilt (HDT) and HDT + CO_2_. (C) Change in MCA_vel_ as a function of the change in P_ET_CO_2_ between Seated and HDT + CO_2_ for SNP+ (filled, n = 4) and SNP− (open, n = 4) groups. Linear regression slopes for the SNP+ (dashed, r^2^=0.7256) and SNP− (dotted, r^2 ^= 0.5077) groups were not significantly different.

**Figure 7:**
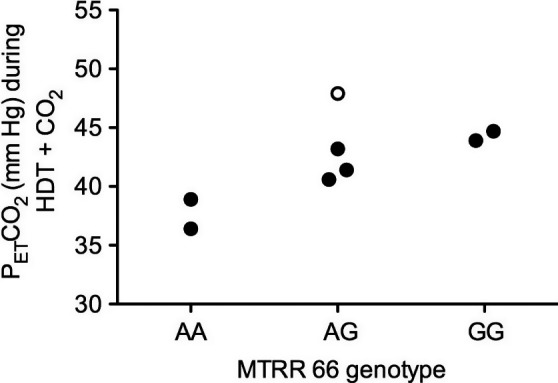
P_ET_CO_2_ during HDT + CO_2_ by MTRR 66 genotype. The subject indicated by the open circle had a B‐12 deficiency which could produce a similar phenotype as the GG subjects.

When classified by genotype group, the change in P_ET_CO_2_ from Seated to HDT + CO_2_ was significantly greater in SNP+ than in SNP− subjects (*P *<* *0.01) (Fig. 6A). However, there was no functional difference in the percentage change in MCA velocity from Seated to HDT or Seated to HDT + CO_2_ (Fig. 6B). Cerebrovascular reactivity appeared similar between groups as the slope relating the change in MCA_vel_ as a function of the change in P_ET_CO_2_ from Seated to HDT+CO_2_ was not different between groups (Fig. 6C). P_ET_CO_2_ data were also plotted by the exact MTRR 66 genotype of each subject (Fig. 7), but statistical analysis was not conducted to determine differences in P_ET_CO_2_ based on the exact MTRR genotype because two of the groups contained only two subjects.

### Regression model

A multiple regression model significantly predicted P_ET_CO_2_ from the number of risk alleles for MTRR and vitamin B12 status (methylmalonic acid), *F*(2, 5) = 14.80, *P *=* *0.0079, *r* ^2 ^= 0.86. P_ET_CO_2_ is equal to 33.06 + 3.03(MTRR) + 0.036(methylmalonic acid), where MTRR is coded as the number of G alleles and methylmalonic acid is measured in nmol/L. Both variables added significantly to the prediction, *P *<* *0.05.

## Discussion

This study investigated (1) vascular changes in the head and eye, (2) IOP, nICP, and the resulting TLPD, (3) ocular structural outcomes, and (4) possible genetic contributions to the spaceflight analog of 6° head‐down tilt combined with acute exposure to mildly elevated CO_2_ concentrations similar to those occurring on the ISS in healthy subjects. The main finding was that although HDT led to baroreflex‐mediated changes in blood flow in response to changes in hydrostatic pressure, an increase in nICP, and a decrease in TLPD, the addition of 0.5% CO_2_ during the HDT + CO_2_ did not augment these changes. However, there were no ocular structural changes detected across the three conditions. Additionally, the genetic analyses helped describe trends or differences in IOP, nICP, and P_ET_CO_2_ responses to tilt and CO_2_ exposure, but these findings should be viewed with caution given the small number of subjects studied. These observations suggest that SNPs in the one‐carbon pathway may contribute to individual differences in the response to some factors believed to be associated with spaceflight‐induced ocular changes.

For decades HDT has been used to induce a headward fluid shift as an analog for studying the physiological responses to weightlessness during spaceflight (Hargens and Vico 2016); however, the ocular structural and functional changes that develop during long‐duration spaceflight have not developed during up to 70 days of HDT bed rest (Taibbi et al. 2013, 2016). It is conceivable that duration of exposure may play a significant role and longer bed rest durations may lead to changes similar to those observed during long‐duration spaceflights. However, it is also possible that some factor not simulated by HDT alone may cause physiological responses that contribute to the ocular changes that develop during long‐duration spaceflight. In this study we sought to identify subtle anatomical and physiological changes associated with the combination of HDT + CO_2_ that may precede the development of ocular changes in astronauts.

### Mild hypercapnia

Mild elevation of ambient CO_2_ on the ISS has been associated with reported headaches in otherwise healthy astronauts (Law et al. 2014) leading to the speculation that CO_2_‐induced cerebral vasodilation, coupled with decreased venous drainage resulting from the loss of the hydrostatic fluid gradient draining the brain, may contribute to the development of ocular findings. We measured P_ET_CO_2_ and cerebral blood flow velocity to determine if a mild increase in inspired PCO_2_ would (1) significantly increase arterial CO_2_ levels as measured by P_ET_CO_2_, (2) if this increase would lead to a significant increase in blood flow in the head and eye, and (3) ultimately further increase ICP which has been hypothesized to be a contributing factor to ocular changes during long‐duration spaceflight.

The 2.7 mmHg increase in P_ET_CO_2_ during HDT was likely caused by the mild hypoventilation resulting from the cephalad shift of abdominal contents (Weissman et al. 1982; Mannix et al. 1984), as the inspired PCO_2_ was not different between Seated and HDT. During HDT + CO_2_ we delivered 7.5 mmHg CO_2_ (1%) to our subjects, which resulted in a further 1.7‐mmHg increase in P_ET_CO_2_ relative to HDT breathing room air. This is slightly higher than the 0.7 mmHg (range: 0.2–1.4 mmHg) increase in directly measured arterial PCO_2_ reported in seated subjects breathing 1% CO_2_ (Ellingsen et al. 1987a,b), but may reflect differences due to posture or time of hypercapnic exposure, or differences related to our assessment of P_ET_CO_2_, which typically gives higher values than direct arterial PCO_2_ measurements. Prisk et al. (1995) reported P_ET_CO_2_ data from crewmembers before, during, and after two short‐duration spaceflights. Before flight, moving from the standing to the supine position increased P_ET_CO_2_ ~3 mmHg. During the first 7‐day mission P_ET_CO_2_ did not increase relative to the preflight standing value, yet P_ET_CO_2_ of subjects on the second 14‐day mission, when ambient inspired PCO_2_ was ~2.3 mmHg, increased ~4 mmHg. The authors concluded that it was unclear whether the elevated ambient PCO_2_ or the microgravity environment led to this mild elevation in P_ET_CO_2_. This same group reported P_ET_CO_2_ during long‐duration spaceflight when the ambient PCO_2_ was ~4 mmHg (Prisk et al. 2006). Relative to the preflight seated P_ET_CO_2_ of 36.7 mmHg, P_ET_CO_2_ on the ISS was slightly elevated at 39.0 mmHg, but nearly identical to the preflight supine P_ET_CO_2_ of 39.7 mmHg. Thus, the headward shift of abdominal contents during weightlessness likely contributed to the small increase in P_ET_CO_2_, whereas the mild elevation of ambient PCO_2_ on the ISS was likely too small to increase P_ET_CO_2_ when measured during long‐duration spaceflight.

More recent data from nine ISS astronauts showed a ~6 mmHg increase in P_ET_CO_2_ during long‐duration spaceflight relative to the preflight seated position (Hughson et al. 2016). While at least part of this increase resulted from the headward shift of abdominal contents and mild hypoventilation, it is unclear why these values were greater than those previously reported (Prisk et al. 2006). Furthermore, it is unknown whether an absolute P_ET_CO_2_ of 42.1 mmHg during spaceflight would lead to physiological consequences related to cerebral or ocular blood flow, as these authors did not collect cerebral or retinal blood flow velocity measurements. In this study, we report an identical P_ET_CO_2_ of 42.1 mmHg during HDT + CO_2_, which was insufficient to elicit ocular changes or any systemic vasodilation, despite an inspired PCO_2_ greater than twice that recently reported from the ISS (Hughson et al. 2016).

### Cardiovascular responses

It is well accepted that the cardiovascular system rapidly responds to changes in posture, but it currently is unclear how hemodynamic responses contribute to changes in ocular structure and function during spaceflight. It has been hypothesized that during HDT, increases in arterial blood flow may increase cerebral blood volume and consequently ICP.

In this study, head‐down tilt increased stroke volume and reduced HR, presumably due increased venous return and a baroreflex response, while MAP and cardiac output were maintained across all three conditions. However, HDT increased the cerebral arterial pressure relative to pressure in the seated position through changes in hydrostatic pressure. This resulted in a 20% increase in MCA_v_ during the HDT condition. During HDT + CO_2_ there was no further change in MAP or diastolic blood pressure relative to HDT, indicating that any difference in MCA blood flow velocity between HDT and HDT + CO_2_ would reflect changes in vascular reactivity to CO_2_ rather than result from a change in arterial pressure. That MCA_v_ was the same during HDT and HDT + CO_2_ conditions suggests the mild hypercapnia did not induce cerebral vasodilation. Similar to our initial hypothesis, Tymko et al. (2015) also speculated that superimposing increased arterial pressure from severe HDT to −90°, with vasodilation due to increases in CO_2_, would result in a greater cerebral blood flow velocity response. Using a rebreathing protocol across severe head‐up and head‐down tilt positions they found cerebrovascular CO_2_ reactivity was maintained across severe tilt angles. Our study employed steady‐state inspired levels of CO_2_ and a less severe tilt angle, but demonstrated similar findings.

The decrease in common carotid artery blood flow during HDT appears to have been driven by the fall in HR and has been demonstrated previously in the internal carotid artery (ICA) using magnetic resonance imaging during 12°HDT (Kramer et al. 2017). In that study breathing 0.5% CO_2_ did not alter the ICA blood flow, but 3% CO_2_ returned it to supine levels. In the current study breathing 1% CO_2_ returned CCA blood flow to levels not different from Seated. Normalizing CCA flow by MAP suggests an increase blood flow resistance occurs between Seated and HDT (0.136–0.140 mL·min^−1^·mmHg^−1^) that is returned to Seated levels during HDT+CO_2_ (0.134 mL·min^−1^·mmHg^−1^). Because a similar pattern did not occur in the MCA_v_, it is possible that blood flow splitting between the internal and external carotid arteries may be differentially regulated in response to mild levels of hypercapnia during HDT. However, using severe 90° HDT Geinas et al. (2012) found no difference in common carotid and external carotid artery blood flow. Future work is needed to determine if blood flow regulation differs between internal and external carotid arteries during a prolonged headward fluid shift.

Because of the change in posture between conditions, the arterial pressure within the central retinal and middle cerebral arteries increased due to the increase in fluid column pressure and this is reflected by the increase in blood flow velocity during the HDT condition. In the central retinal artery the increase in blood flow velocity during HDT was similar to previous reports (Sirek et al. 2014), but no further increase during HDT + CO_2_ occurred. It has been suggested that an increase in CSF pressure surrounding the optic nerve could compress the central retinal artery, which also lies within the optic nerve sheath, and that a change in central retinal artery blood flow velocity could reflect changes in ICP (Sirek et al. 2014). We normalized the change in blood flow velocity for the change in hydrostatic pressure during HDT and demonstrate that there was no change in conductance index, indicating the mild hypercapnia did not induce vasodilation. Because these data demonstrated no change in retinal vascular conductance across all three conditions, and our measures of nICP were not different between the two HDT conditions, it appears that pressures external to the common retinal artery did not alter the blood flow velocity either.

### IOP, ICP, & TLPD

The role of ICP and TLPD has been investigated to describe and understand the pathogenesis of optic disc neuropathies as various methods for noninvasively assessing ICP have improved sensitivity and specificity (Fleischman and Allingham 2013; Siaudvytyte et al. 2015; Morgan et al. 2016). Accordingly, NASA has also gained interest in assessing these parameters to potentially help explain the development of ocular changes in astronauts (Berdahl et al. 2012). As recently hypothesized, a dysregulation of the pressure balance between the IOP and the ICP across the lamina cribrosa may lead to the development of optic disc abnormalities (Jonas et al. 2015a) such as the optic disc edema in astronauts on the ISS.

In this study, we used a model for noninvasively estimating ICP that is based on the individual's TCD MCA waveform morphology, arterial pressure, and ECG. Previously this model successfully detected a rapid increase in ICP during a 5% CO_2_ inhalation (Asgari et al. 2011), suggesting that this model has the sensitivity to detect changes in ICP resulting from increased cerebral blood flow. However, this is the first time we have applied this model to a positional change with and without mild hypercapnia. During HDT, nICP increased by ~7 mm Hg, which is similar to that reported by Lawley et al. (2017), who directly measured ICP using a pressure transducer inserted into the Ommaya reservoir of asymptomatic patients during Seated and 6° HDT positions. Furthermore, Eklund et al. (2016) measured ICP by lumbar puncture in subjects while they were seated and during 9° HDT, reporting a slightly higher ICP than our estimated nICP measured during 6° HDT. In our investigation, however, the combination of the headward fluid shift due to HDT with mild hypercapnia did not further augment nICP. Using the Vittamed noninvasive ICP device Marshall‐Goebel et al. (2017) also demonstrated no difference between ICP during 12° HDT breathing room air and while breathing 1% CO_2_ for 3.5 h, although the resulting P_ET_CO_2_ were not reported.

We also demonstrated an increase in IOP with HDT as others have previously shown (Mader et al. 1990; Chiquet et al. 2003; Macias et al. 2015) and a small, but statistically significant further change during HDT + CO_2_. This finding confirms previous observations of increased IOP during increases in arterial or end‐tidal CO_2_ (Kielar et al. 1977; Petounis et al. 1980; Hvidberg et al. 1981; Awad et al. 2009). Despite these significant increases, all measures of IOP were within normal limits and the measured changes were not clinically relevant, particularly for healthy subjects. Paradoxically, some studies have demonstrated no change (Hosking et al. 2004) or a decrease in IOP (Harris et al. 1996; Kergoat and Faucher 1999) in response to 5–6% inspired CO_2_. This may reflect changes in sympathetic stimulation and resulting increases in catecholamine concentrations, which following exercise leads to an increase in the area of Schlemm's canal and could contribute to reductions in IOP (Yan et al. 2016). Together, our measures of IOP and nICP suggest that HDT and HDT + CO_2_ cause similar reductions in TLPD from Seated. These data suggest that the change in TLPD across the lamina cribrosa due to a headward fluid shift is not augmented further during acute exposure to mild elevations in CO_2_.

### Optic nerve sheath diameter

Because the optic nerve is bathed in cerebrospinal fluid (CSF), ICP elevations can be transmitted to the subarachnoid space surrounding the optic nerve, causing enlargement of the ONSD (Hansen and Helmke 1996). Magnetic resonance imaging assessment of the ONSD on Earth after spaceflight demonstrated a distended ONSD (Mader et al. 2011; Kramer et al. 2012), and ultrasound assessments of ONSD during spaceflight support these reports (Mader et al. 2013). Despite the acute timeframe of the current study, ONSD has been shown to respond within 1 min, and to remain stable over the subsequent 5 min in response to a 10‐mm Hg fall in P_ET_CO_2_ (Kim et al. 2014). This suggests that our measurements, which occurred ~25 min after the onset of HDT or HDT + CO_2_, were made following a sufficiently long period of time after the intervention to detect distension of the ONSD in the subarachnoid space. Our data support the influence of the headward fluid shift associated with HDT, which occurs during weightlessness in space, as a contributing factor to an increased ONSD; however, an acute mild elevation in ambient CO_2_, similar to that on the ISS, did not further increase ONSD.

**Figure 8:**
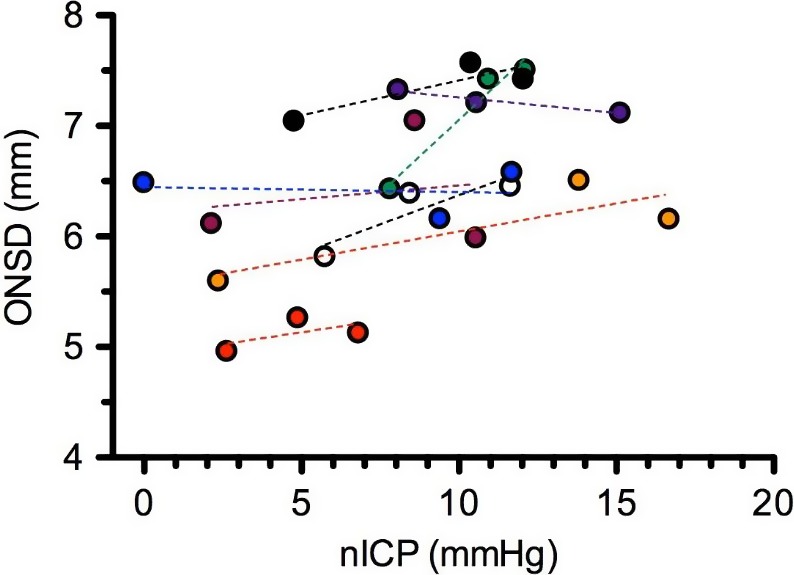
Optic nerve sheath diameter (ONSD) as a function of nICP during each condition for all eight subjects. Dashed lines represent linear regression of data for each subject.

Despite the nonpathologic range of ICP, our ONSD data showed a response similar to our nICP data during HDT and HDT + CO_2_, respectively. However, plotting ONSD as a function of nICP for all subjects in each of the three conditions reveals the intersubject variability in both nICP and ONSD (Fig. 8). We interpret this relationship to suggest ONSD by itself does not provide sufficient sensitivity to differentiate changes in ICP within the physiologic range measured in this study.

### Ocular edema

The implementation of OCT imaging before, during, and after spaceflight has revealed ocular structural changes including optic disc edema and choroidal folds (Mader et al. 2011). We used the Spectralis HRA + OCT AutoRescan feature and the new APS system, which centers a radial scan pattern over the fovea‐to‐Bruch's membrane opening center axis (Chauhan and Burgoyne 2013), to identify whether subtle changes in fluid accumulation surrounding the optic disc would be observed during HDT with the addition of 1% CO_2_. Our analysis did not reveal significant changes in BMO area or minimum rim width, suggesting no signs of early optic disc edema. Thus, it was not surprising that there was no evidence for RNFL thickening or choroidal folds in images obtained during any condition. A 1‐h exposure to HDT with or without mild hypercapnia does not appear to be a sufficient stressor to elicit significant physiological changes that may contribute to the etiology of long‐term ocular structural changes.

Advancements in spectral domain‐OCT imaging, including Enhanced Depth Imaging, have improved the clarity of the choroid‐sclera border posterior to the retina, providing opportunities to assess changes in choroidal thickness using OCT (Yiu et al. 2014). Choroidal blood flow increases during HDT (Shinojima et al. 2012) and in response to high levels of CO_2_ (Friedman and Chandra 1972; Delaey and Van De Voorde 2000) as well as changes in ocular perfusion pressure or IOP (Polska et al. 2007). Thus, choroidal engorgement has been hypothesized to contribute to the hyperopic shift that develops in some astronauts. Our two observers conducted masked segmentation of the choroid‐sclera border and averaged the choroid thickness across a 3‐mm section centered under the fovea; the average difference between the two observers was 4.0 ± 2.3% (mean ± SD). During both HDT and HDT+CO_2_ we detected a significant increase, relative to Seated, in subfoveal choroid thickness similar to what others have reported despite different analysis procedures (Anderson et al. 2016). It seems unlikely this change in choroid thickness contributed to the increased IOP measured during HDT and HDT + CO_2_ since IOP increased further during HDT + CO_2,_ but choroid thickness did not. Others have suggested that increased arterial PCO_2_ does not alter choroid thickness (Ozcimen et al. 2015) although they did not study the possible synergistic stimulus of HDT during hypercapnia, nor did they report P_ET_CO_2_ values to clarify the amount of the hypercapnic stimulus. Had significant vasodilation developed in arteries feeding the choroid we would have expected the lack of autoregulation in the choroid to permit vascular engorgement and potentially alter visual acuity from a resulting hyperopic shift. Due to the minor changes in choroid thickness it was not surprising to detect no changes in visual acuity. Despite this, our data suggest that acute mild hypercapnia during HDT does not increase choroid thickness further.

### Genetic and biochemical variables

We have previously reported a significant 1‐ to 2‐*μ*mol/L difference in homocysteine concentration between astronauts with and without ophthalmologic changes after spaceflight (Zwart et al. 2012). Though not statistically significant, the difference in mean homocysteine concentration between SNP+ and SNP*−* groups in this study was 1.3 *μ*mol/L (*P *=* *0.13), which is within the range of differences observed in the spaceflight study. Furthermore, this is within the range expected for individuals with different SNPs of genes associated with the one‐carbon pathway (Kluijtmans et al. 2003; Davis et al. 2005; Crider et al. 2011). Interestingly, even with only eight subjects, the allele incidence for the five SNPs analyzed in this study was similar to the allelic frequency for the same SNPs analyzed in a previously published study of 49 astronauts (Zwart et al. 2016).

To connect CO_2_ exposure, genetic SNPs profile, and physiological changes that may contribute to the ocular changes during long‐duration spaceflight, we hypothesized that grouping subjects based on these SNPs would reveal differences in physiological responses during the HDT+CO_2_ condition. The significant difference in the nICP response to HDT between SNP+ and SNP− groups suggests 1‐carbon genetics may be a predisposing risk factor for physiological responses related to a headward fluid shift. This study tested the hypothesis that exposure to 1% CO_2_ during HDT would lead to changes in cerebral and/or ocular blood flow, which would contribute to an augmented ICP. Because 1‐carbon genetics appears to be a risk factor for the development of certain ocular changes, we further hypothesized that those physiological changes that develop during the HDT + CO_2_ would be greater in those subjects with SNP+ alleles. While both SNP+ and SNP− groups demonstrated a similar TLPD during the three phases of the study, the strategy by which physiological responses develop in these groups may differ and the long‐term consequences of these differences is unknown. There was no difference in cerebral blood flow response to HDT or HDT + CO_2_ between SNP+ and SNP− groups, which may explain why we did not detect a significant difference between groups in the response of nICP to HDT+CO_2_. However, interpretation of these data are challenging due to the small number of subjects. These pilot data warrant future studies to determine whether physiological responses to a headward fluid shift can be predicted based on genetic polymorphisms.

Some have speculated that elevated ambient CO_2_ levels on the ISS may contribute to the development of ocular changes in astronauts during long‐duration spaceflight (Law et al. 2014). Ventilatory and cerebrovascular sensitivity to changes in arterial CO_2_ levels vary between subjects, resulting in different arterial PCO_2_ levels for a given inspired PCO_2,_ which we demonstrated from the P_ET_CO_2_ measures for our group of subjects as a whole. Our measure of P_ET_CO_2_ as a surrogate for arterial PCO_2_ demonstrated an increase during HDT that was augmented further during HDT + CO_2_. This change in PCO_2_ from Seated to HDT + CO_2_ was significantly greater in SNP+ than in SNP− subjects, suggesting a possible interaction between genetic polymorphisms, inspired or ambient PCO_2_, and resulting arterial PCO_2_ levels. Furthermore, statistical modeling, using a model similar to one reported previously for predicting ophthalmologic changes after spaceflight, demonstrated that both B‐vitamin status (vitamin B12) and genetics (number of MTRR risk alleles) significantly predicted the P_ET_CO_2_ during the HDT + CO_2_ condition. However, a link between alterations in the one‐carbon metabolism pathway and ventilatory sensitivity to CO_2_, which could explain this difference in P_ET_CO_2_, is unknown. Separation of subjects on the basis of their MTRR 66 genotype suggests that a potential protective factor against elevated P_ET_CO_2_ is the AA genotype, which also seemed to protect against the development of cotton wool spots or choroidal folds in astronauts (Zwart et al. 2016). Future work should focus on identifying whether these or other genetic polymorphisms and B‐vitamin status can be used to predict whether certain subjects are more susceptible to developing hypercapnia in the face of mildly elevated ambient CO_2_ levels, and on determining what role this may play in the development of ocular structural and functional changes.

### Limitations

First, direct ICP measures were not obtained in this study, and we lack data regarding the accuracy of the adopted nICP among normal subjects. The approach was developed and validated in brain‐injury patients, and we recognize that the relationship between the nICP model and direct measures of ICP may differ between patients and healthy subjects. However, the range of nICP for the subjects in this study was physiologically plausible at both the HDT and seated positions based upon previous reports from invasive studies. The increase in nICP from Seated to HDT was similar to recently reported ICP values measured from lumbar puncture in subjects seated, supine, and undergoing 9° HDT (Eklund et al. 2016), as well as directly measured ICP in patients with implanted Ommaya reservoirs during 6° HDT and brief periods of weightlessness during parabolic flight (Lawley et al. 2017). Although these results indirectly support the validity of the nICP approach used in the current study, direct validation of nICP with invasive ICP data from normal subjects should be considered in future research.

Second, our outcome measures could not be taken concurrently at the end of each condition; rather, the testing procedures had to be performed sequentially. However, our predefined order of operations ensured that all contact procedures, such as ocular ultrasound, were performed after the noninvasive tests, such as IOP and Spectralis HRA+OCT imaging.

Third, our experimental setting evaluated only the acute responses to mild hypercapnia during HDT. This may explain why changes in TLPD, which are hypothesized to contribute to the development of optic disc edema during long‐duration weightlessness, may not have resulted in optic disc edema during our study. While chronic exposure to mild hypercapnia during HDT has not been studied in humans, Wistar rats chronically maintained in 10% CO_2_ for 21 weeks had ICP that was not different from the normoxic control group in which an acute exposure to 10% CO_2_ doubled ICP (Kondo et al. 1999). While intriguing, this degree of hypercapnia and differences between quadrupedal and bipedal locomotion make interpretations of these data with respect to astronauts in space difficult.

Fourth, while the subjects wore the facemask throughout all three conditions, during room air conditions they were not connected to the breathing reservoir used during the HDT + CO_2_ condition. While this would have made it possible to blind the HDT + CO_2_ condition from the subjects, we do not believe this significantly impacted our results as no subjects reported differences in CO_2_ symptoms between the HDT and HDT + CO_2_ conditions.

Finally, this was the first time one‐carbon pathway genetic polymorphisms were analyzed in test subjects participating in physiological testing to understand mechanisms that may contribute to the ocular structural and functional changes that develop during long‐duration spaceflight. Therefore, we could not prospectively identify subjects with certain genetic polymorphisms for inclusion in this study. With only four subjects in each subgroup caution should be employed when interpreting group‐wise comparison results.

## Conclusions

In 2011 NASA documented the initial cases of ocular abnormalities in long‐duration astronauts and termed the constellation of findings the Visual Impairment and Intracranial Pressure (VIIP) syndrome. Due to mounting evidence suggesting pathologically elevated intracranial pressure is not likely occurring during spaceflight, some have used other naming conventions, such as Microgravity Ocular Syndrome or Astronaut Ocular Syndrome. To reduce the emphasis on intracranial pressure, the medical operations community within NASA is in the process of renaming VIIP to the Spaceflight Associated Neuro‐ocular Syndrome (SANS).

In this study, the combination of an acute headward fluid shift with inspired CO_2_ twice as high as the average levels on the ISS did not cause ocular structural or functional changes. Our noninvasive estimate of ICP, using a novel machine‐learning algorithm, and the addition of mild hypercapnia during HDT, did not further increase nICP. During acute HDT IOP, ONSD, and choroid thickness increased, but with the exception of IOP, mild hypercapnia did not further increase these ocular changes from the baseline Seated condition. Together, these data suggest the combination of mild hypercapnia with an acute headward fluid shift does not augment physiological factors hypothesized to contribute to ocular changes during spaceflight. However, grouping of subjects by genetic polymorphisms may provide promising insight into understanding the individual variability in many physiological outcomes that develop during spaceflight, including greater susceptibility to increased arterial PCO_2_ levels, potentially increasing the risk for symptoms related to CO_2_ exposure. Future studies are needed to investigate the effects of chronic exposure to mild hypercapnia with a headward fluid shift to understand whether the elevated CO_2_ levels on the ISS may contribute to development of ocular changes. These studies should continue collection of genetic and nutritional status data to validate the preliminary findings presented here and identify novel mechanisms to better understand the development and progression of ocular structural and functional changes during long‐duration spaceflight.

## Conflict of Interest

None declared.
